# Time to move to open access

**DOI:** 10.1093/eurpub/ckaa231

**Published:** 2021-01-27

**Authors:** Peter Allebeck, Dineke Zeegers Paget, Iveta Nagyova

**Affiliations:** 1 Department of Global Public Health, Karolinska Institutet, Stockholm, Sweden; 2 European Public Health Association, Utrecht, The Netherlands; 3 Department of Social and Behavioural Medicine, Faculty of Medicine, European Public Health Association, PJ Safarik University, Kosice, Slovakia

The publishing landscape is rapidly shifting. A few decades ago, almost all journals were based on subscriptions, paid by libraries, universities and research institutes. During this century, we have seen a strong growth of open access journals, where the cost of publishing is covered by authors.

There is increasing awareness in society that results from publicly funded research should be made freely accessible. Scientists, policymakers, journalists and other stakeholders should be able to access the latest scientific information without having to pay large costs to the publishers.

There is now strong international momentum to increase the transition to open access, e.g. through the OA 2020 initiative.^1^ In Europe, there has been intense discussions on the ‘Plan S’,^2^ signed by a number of research funders in Europe and some other countries, including major players such as the Wellcome Trust and the Bill and Melinda Gates Foundation. This collaboration between a number of major research funders, supported by the European Commission, has given incentive to publishers to review business models and accomodate to open access.

Many research funders and institutes now require scientists to publish research results through open access. The classical subscription journals have accommodated this by creating ‘hybrid journals’, with an extra cost for those who wish to publish open access. For many years, this journal has been, and still is, a hybrid journal, but with an increasing level of papers published open access, now around 25%. The hybrid model is not considered an optimal solution for financing open access, since it implies payment for both subscription and publishing.

One long-term solution for financing scientific publication is through ‘read-and-publish agreements’, whereby a country, or a consortium of universities or research funders, pay an overall sum to publishers allowing both access to reading and to publish open access. *The European Public Health Association*’s (*EUPHA*’s) policy has been to advocate for read-and-publish agreements. Such agreements are already in place in several countries.

*EUPHA* has also advocated for maintaining the learned societies’ role in the running of scientific journals. Many ‘pure’ open access journals are just catalogues of scientific manuscripts, whereas societies want to include editorials, viewpoint papers, commentaries, society news etc., in their journals. *EUPHA* is determined to keep the *European Journal of Public Health* (*EJPH*) as a society journal, combining high level scientific publication with policy papers, debates, and public health news. This is an important part of *EUPHA*’s knowledge translation policy, implying a priority for the association to strengthen public health science, policy and practice in Europe.

During the past year, *EUPHA* and its members have discussed a possible shift for the *EJPH* to a complete open access journal. Overall, reactions have been positive, although there remains concern regarding weaker institutions and authors without funding to pay for open access.

Understandably, the cost for publishing, APC (Article Processing Charge) is a point of concern, It should be pointed out that the overall goal with plan S and shift of payment streams is that publishing costs should not be covered by individual authors, but by public bodies such as research funders, universities or libraries, by funding mechanisms that already are in place in many countries. The analyses performed on national as well as global level all point to the fact that ‘there is money in the system’, i.e. costs paid today by libraries and universities to publishers for subscriptions, are higher than what all APCs would amount to.^3^ In the move to open access, *EUPHA* will find a mechanism to allow possibility to publish in this journal also for those who are not covered by some type of funding for publication or who are not part of read-and-publish agreements.

Beginning in January 2022, this journal will be published fully open access. The current structure with six issues per year, and with editorials, viewpoints and European public health news, will remain. Paper copies that are currently being distributed in big packs all over Europe, will no longer exist, to the benefit of the environment. Certain supplements, for which paper copies are important for targeted dissemination, can still opt for paper copies. Thus 2021 will be a transition year, when we publish all papers accepted on the current agreement, whereas papers submitted from May 2021 will be published under the new agreement.

With this move to open access, *EUPHA* and the *EJPH* follow what is now a strong momentum to make scientific knowledge freely available. By this, we also believe the journal can play an even stronger role for public health science and policy in Europe.

*Conflicts of interest*: None declared.
